# Valuation of Medical Innovation in Orphan Diseases with a Focus on Small Investors and Limited Diversifiable Risks

**DOI:** 10.3390/jmahp14030047

**Published:** 2026-08-05

**Authors:** Mark Nuijten, Pieter van Gelder

**Affiliations:** 1 A2M, 4611 MS Bergen op Zoom, The Netherlands; 2 Section Safety and Security Science, Faculty of Technology, Policy and Management, Delft University of Technology, 2628 BX Delft, The Netherlands; p.h.a.j.m.vangelder@tudelft.nl

**Keywords:** economic valuation, orphan drugs, uncertainty, R&D innovative drugs, real options

## Abstract

This paper assesses the impact of uncertainty for investors on the economic valuation of medical innovation projects for orphan drugs or rare diseases. Conventionally, investor evaluation uses the deterministic discounted cash flow (DCF) method with an appropriate sensitivity analysis that captures some level of uncertainty. In healthcare, and particularly for rare diseases, the levels of uncertainty in financial outcomes (return on investment and net present value (NPV)) are broader than the ones normally captured by the DCF formula. Uncertainties include R&D costs, the approval process (level and timing) for obtaining reimbursement, sales, the production cost, and the failure probabilities of the clinical trial phases, to name a few. Additionally, there is not only one type of investor to consider, but different investors exposed to different levels of risk management of their investment. Our analysis tried to capture those two dilemmas (higher levels of uncertainty and different investor types) in two ways. One way was to identify a better method to enhance the different levels of uncertainty. The real option method of evaluation was proposed instead of DCF. For instance, the real option method better captures the uncertainty of the different phases of product development. The other way is to differentiate the investor types through their level of risk assessment perspectives. Small investors and start-up companies may see more benefit in applying the real option methodology to estimate their NPVs at different time points during product development. In summary, our evaluation identified various types of uncertainty when assessing an investment, along with methods to manage their effect on the economic/financial outcomes of medical innovations. Given the high uncertainty associated with early-stage drug development, such as orphan drugs for rare diseases, the real options approach is preferable to traditional DCF models. The analysis also showed that there is not just a single investor perspective to consider but specific perspectives that enhance the prime use of the real option methodology.

## 1. Introduction

In recent decades, the pharmaceutical and biotechnology sectors have experienced unprecedented growth in the development of innovative therapies, particularly for orphan diseases. Advances in biotechnology have led to groundbreaking therapies such as gene therapy and immunotherapy, which promise significant clinical benefits for orphan diseases.

Immunotherapy is a medical treatment that uses the body’s own immune system to prevent, control, or destroy diseases such as cancer. Instead of directly attacking cancer cells like chemotherapy, immunotherapy helps immune cells recognize and fight abnormal cells more effectively. Examples include monoclonal antibodies, CAR-T cell therapies, and cancer vaccine therapies [1,2]. Gene therapy is a medical technique that modifies or replaces genes inside a person’s cells to treat or cure disease. It works by correcting faulty genes, adding new genes, or turning off harmful genes [3].

The development of orphan-designated drugs has been more challenging and financially less attractive than that of other drugs due to the low prevalence of certain conditions, difficulty in applying randomization and controls in trial design, the geographical dispersion of patients, poorly defined biomarkers, and the lack of healthcare provider experience in diagnosing and treating certain conditions [4].

The economic valuation of medical innovations, particularly those targeting rare and orphan diseases, has become increasingly complex due to the high levels of uncertainty associated with their clinical development and commercialization [5,6]. Accurately assessing the economic value of such R&D projects is critical for attracting investment and guiding strategic decision-making on positioning and pricing. However, traditional approaches, such as the discounted cash flow (DCF) model, often fall short in adequately capturing the high degree of uncertainty and risk inherent in early-stage R&D projects for highly novel treatments like gene therapies and immunotherapies [7,8,9].

DCF models do not sufficiently capture the uncertainty in key parameters, such as clinical trial outcomes, regulatory approval, cost-effectiveness, market access and reimbursement, and market uptake, especially in the context of gene therapy and immunotherapy. Moreover, the timing of clinical R&D projects introduces an additional source of uncertainty in economic valuation that is not captured by the DCF model. Although the discounted cash flow approach incorporates time through discounting via the cost of capital, it does not fully reflect investor preferences for earlier returns. For example, an investor will typically prefer a project that generates sales within one year over another project with a similar economic value but with sales expected only after, for instance, eight years. Hence, the DCF approach is not fully appropriate for decisions regarding investment in innovative drugs, due to the high degree of uncertainty and long-time horizons. This is especially true because start-up biotech firms are usually not profitable during the first eight to ten years [10]. In an economic environment defined by great uncertainty and rapid change, it has become increasingly important to employ valuation methods that appropriately reflect uncertainty, as well as a company’s ability to respond to new information. Real options have emerged as an approach that addresses these challenges more effectively than traditional DCF techniques and can be applied to capture the specific uncertainty associated with the economic valuation of early-phase drug development for orphan diseases [11,12,13].

The objective of this paper is to assess the economic value of medical R&D projects by incorporating uncertainty more explicitly into the valuation process. We focus on economic value from the perspectives of different types of investors and examine how uncertainty influences the economic outcomes for each type of investor depending on investment type and timing. Specifically, we consider the perspective of large investors with diversified portfolios, small investors participating in portfolio funds, small investors investing in individual R&D projects, and the initial founders of start-ups. Special attention is given to highly innovative projects (e.g., immunotherapy or gene therapy) for orphan diseases, which offer a useful framework due to their pronounced uncertainty and unique market dynamics.

## 2. Evaluation Concepts

### 2.1. DCF Concept

The economic value of an innovative R&D project, seen from an investor point of view, is a function of its current and potential future revenues (sales) and expenditures (costs). The DCF approach is based on the present value equation, which yields the time value of money and compounding returns [14]. In the DCF approach, expected project cash inflows and outflows are discounted to their present value using a rate that reflects both risk and the time value of money. These discounted cash flows are then aggregated, and initial investment costs are deducted to calculate the project’s net present value (NPV). According to theory, a project should be accepted if its NPV is positive, as it is expected to enhance shareholder value.

(1) $$NPV=\frac{{CF}_{1}}{{\left(1+r\right)}^{1}}+\frac{{CF}_{2}}{{\left(1+r\right)}^{2}}+\cdots +\frac{{CF}_{n}}{{\left(1+r\right)}^{n}}$$

where

NPV = net present value.

CF = (free) cash flow.

n = the time in years before the future cash flow occurs.

r = cost of capital or discount rate.

We note that the NPV converges to CF/r for n to infinity, with CF being constant over time.

Free cash flows from operations correspond with the revenues from drug sales, and the costs of R&D, production, and marketing. The discounted cash flow method is based on a deterministic approach, and therefore the economic value does not reflect any uncertainty in the values of specific parameters.

### 2.2. CoC

The cost of capital (CoC) is the minimum return that an investor expects for providing capital for a project. The CoC is a separate parameter in valuation which reflects the investor’s risk and corresponds with the required return for the investor. It represents the risk profile of the company and will be used as a proxy for the required rate of return. The same discount rate will be used for cash flows [15]. The CoC should not be based on the expectations at the time of the reimbursement application, but rather on the expectations at the time of investment. The Capital Asset Pricing Model (CAPM) describes the relationship between systematic risk and expected return for assets, particularly stocks, which yields the CoC [16].

Cost of capital = ER*i*

ER*i* = R*f* + beta *i* (ER*i* − R*f*)

where

ER*i* = expected return of investment.

R*f* = risk-free rate.

beta*i* = beta of the investment.

(ER*m* − R*f*) = market risk premium.

For beta > 1, the portfolio is more profitable than the average market.

What is the relationship between DCF and CoC? Cash flows (DCF) can be used to project the expected free cash flows over a specific period (e.g., 5 to 10 years), plus a terminal value at the end to represent all cash flows beyond that projection period. The cost of capital acts as the ‘discount rate’ in the DCF formula. It represents the minimum expected return an investor or company needs to justify an investment, accounting for risk and the time value of money. The formula translates future cash flows (CF) at time period (t) into a present value using the cost of capital (r).

$$Present\;Value=\sum_{t=1}^{n}\frac{{CF}_{t}}{{\left(1+r\right)}^{t}}$$

A higher CoC (more risk) reduces the present value of future cash flows, leading to a lower overall valuation for the business or project.

## 3. Uncertainty Levels

### 3.1. Definitions

In economic valuation, we distinguish two types of uncertainty: (1) uncertainty resulting from statistical distributions of input variables, known as risk; (2) uncertainty resulting partially from assumptions, including unknown future changes.

Modern finance theory describes two different kinds of investor risk: diversifiable risk and undiversifiable risk [17,18]. Non-diversifiable or systematic risk is the risk that the investor cannot eliminate by diversifying their investment portfolio, for example, macroeconomic risks such as the global recession, which cannot be eliminated. If the investor does not have knowledge of the technical risks of failure of an R&D project, the probabilities of registration and subsequent reimbursement, and the forecasts of costs and turnover for a new specific R&D project, as they differ from those for an average R&D project, the unknown values of these parameters can be considered statistical risks, which are diversifiable and therefore do not affect the required return for an investment. For example, if for a new innovative medicine with a new mechanism of action there is a ‘known’ expectation that the probabilities, costs, and sales will be different from the average values in the pharmaceutical market, the investor will incorporate these different values into the DCF model, which will result in a different NPV.

The NPV for innovative treatments like immunotherapy and gene therapy is likely different than non-orphan drugs lower due to the following: (1) different, and probably higher, probabilities of failure for each phase in R&D program, positive registration, and reimbursement; (2) different, and probably higher, costs of R&D, manufacturing, and marketing; and (3) lower sales (e.g., smaller patient population, restricted use, ‘managed entry agreements’, and substantial price discounts). But even after correction for these different average values, these parameters (probabilities of failure, costs, and sales) can also have a larger spread, which means a higher risk in relation to the expectations for innovative medicines, such as immunotherapy and gene therapy.

However, these higher standard deviations do not affect the average deterministic NPV based on the DCF method. Therefore, one could argue that greater uncertainty, e.g., the risk due to higher standard deviations, is diversifiable for the investor: if the investor spreads the uncertainty over a large portfolio of R&D investments with a similar NPV, the average NPV will be approximately the deterministic NPV, even if the standard deviations vary between different R&D projects. However, if the parameters in the economic valuation have *a priori* higher standard deviations, the investor, who is generally risk-averse, will incorporate any higher standard deviations by including a premium in the CoC.

The usual average risk, e.g., standard deviation (SD), for normal innovative drugs is already captured by the investor in the CoC and therefore, for very innovative drugs like immunotherapy and gene therapy, only the increase in SD leads to an additional premium in the CoC.

### 3.2. Costs

R&D cost forecasting may be derived from the literature. The average costs of developing a new innovative drug are US$704 million [19], but there is a wide range, as recently reported by Rennan, from 318 million to $2.8 billion [20]. The investor may fine-tune these average R&D costs to specific R&D costs for an orphan drug. Recruiting enough patients may require more effort and time, making it more expensive [21]. There are also arguments that R&D costs may be lower because regulatory authorities may accept clinical trials with smaller sample sizes and these authorities may not always require data from phase III clinical trials. Berdud, drawing on estimates from Mestre-Ferrandiz, suggested that the research and development costs for an orphan drug are roughly 23% of those associated with a non-orphan drug [8,21]. This figure is broadly consistent with other studies, which indicate that phase III trial costs for orphan drugs are approximately 25% of the costs incurred for non-orphan drugs [22,23,24]. Berdud also estimated that R&D costs represent 34% of total lifecycle costs [21]. On the other hand, the manufacturing process for breakthrough drugs, such as immunotherapy cell therapy and gene therapy, may be significantly more expensive than that for a new generation of drugs with a similar mechanism of action because of more complex procedures, involving specialized instruments, reagents, stringent quality control measures, and expensive materials and equipment [5,6,25,26]. As a result, the overall manufacturing burden for these innovative therapies is significantly higher than for traditional small-molecule or even standard biologic drugs; manufacturing costs may exceed an estimated 100,000 USD per patient [27,28].

The marketing costs associated with first-in-class innovative drugs are often higher than those for follow-on products, as companies must invest significantly in educating the market and building relationships with clinical investigators and other key stakeholders. Because these therapies introduce a novel mechanism of action, healthcare providers frequently lack an established framework to evaluate them, requiring substantial efforts in scientific exchange, key opinion leader engagement, and disease-state education [29,30]. Moreover, stakeholder engagement, particularly with clinicians, payers, and professional networks, is a central component of pharmaceutical marketing strategies, further increasing the resources needed during launch.

The estimates of probability of success for phase I, phase II, and phase III are between 49% and 75%, 30% and 48%, and 50% and 71%, respectively [8,9]. Recent data for the period between 2014 and 2023 show that the likelihood of approval (LOA) for a new drug is now just 6.7% [31]. The main difference is the lower probability of success of 28% for phase II, whereas probabilities for phase I (47%) and phase III (55%) are near a one-in-two likelihood, and the probability of regulatory approval after filing is 92%. For rare disease drugs, the LOA has been reported to be 17.0%, with probabilities of success of 67.4% (phase I), 44.6% (phase II), and 60.4 (phase III) [31]. The largest difference was in the probability of success for phase II (44.6% for rare disease vs 28.9% overall). Overall, the cumulative clinical success rate appears to have decreased over time. Despite the belief that better science may lead to more successful new drugs, important technological challenges for innovative therapies, especially for immunotherapy, may actually lead to higher failure rates as science advances. Failure rates appear to have increased over time and have fluctuated across stages of development. First, regulators are becoming more risk-averse and may be more reluctant to approve some drugs. Secondly, innovative drugs have novel mechanisms of action or clinical endpoints that may be less clear-cut. On the other hand, new opportunities may help offset increased failure rates; for example, improved preclinical screening can enable earlier project termination.

Overall development time (phases I–III) used to be around 6.5 years on average. Phase III trials tended to be the longest development phase, although the most recent work suggests that development times for phases II and III are now similar [8]. Recent data from the 2011–2020 period indicate that it takes approximately 10.5 years for a drug to successfully progress from phase I development to regulatory approval, which includes 2.3 years for phase I, 3.6 years for phase II, 3.3 years for phase III, and 1.3 years for the regulatory process [31]. Development timelines differ considerably according to numerous factors, including disease area and indication, the quality of clinical trial design practices, and patient availability. Demirci reported that the typical clinical development time for a novel orphan-designated drug was 7.2 years [4]. For a typical study on a novel orphan-designated drug, the durations of phases I, II, and III were 5.4 years, 5.4 years, and 2.7 years, respectively. Recent data by Brown reports that the median clinical development time for innovative drugs was about 8.3 years, with averages often reported around 9.1 years depending on the cohort and methodology [32]. Drugs, particularly orphan drugs, that receive accelerated approval tend to complete clinical development substantially faster, by approximately 3 years, whereas drugs granted breakthrough designation also show accelerated development timelines, shortening development by about 1.3 years. On the other hand, orphan drugs often tend to take longer to develop clinically on average (around 1.5 years) [32]. Even though these drugs often involve smaller clinical trials, development can still be slowed by difficulty finding and recruiting enough patients, limited knowledge about how the rare disease naturally progresses, and the need to create new ways to measure whether the treatment works (new clinical endpoints).

From an investor’s perspective, there is an argument against using actual costs for research and development as well as other expenses (manufacturing, marketing), as these costs are not yet available at the start of the R&D program. Instead, investors must rely on forecasts based on alternative sources.

According to the principles of economic valuation, the R&D costs of unsuccessful programs should be considered. Therefore, allocating the costs of R&D failures to successful drugs that obtain approval from the European Medicines Agency (EMA) or the U.S. Food and Drug Administration (FDA) is an important element of valuation [10,33]. Therefore, probabilities of failure of the clinical trial phases (phase I, II, and III) lower the potential cash flow expected from an investment [9,19].

### 3.3. Sales

The probability of a positive reimbursement decision by national health authorities does not only depend on the registration data for efficacy and safety, but also on additional critical determinants, e.g., cost-effectiveness and budget impact, and their weight varies per country [9]. For example, the incremental cost-effectiveness ratio (ICER) is considered a critical outcome for a reimbursement assessment in the United Kingdom, whereas budget impact is currently more important in France and Germany. The ICER is the additional cost for a life year gained in perfect health, or a quality-adjusted life year (QALY). The ICER reflects the willingness to pay for a gain in one extra QALY, which depends on a country-specific threshold, e.g., €80,000 per QALY in the Netherlands. The probability of reimbursement for innovative therapies may be lower because of the huge ICERs, e.g., the ICER for Zolgensma was €138,875/QALY in The Netherlands [34]. The ICER often guides price negotiations, which may result in an 80% discount to reduce the ICER below the acceptable threshold [9].

The size of the market for orphan drugs is usually quite small because of the limited number of potential patients. For example, the resulting average patient population per 50,000 inhabitants for an orphan drug was 2.91 in NICE appraisals, compared with 102.57 for non-orphan drugs [21]. Thus, the average patient population for orphan drugs was only about 3% of that for non-orphan drugs. However, the development costs of orphan drugs are 23% of those associated with a non-orphan drug [8,21]. Consequently, orphan drugs are expected to be priced substantially higher than other drugs, as development costs must be recouped from a much smaller patient population. This higher pricing often results in ICERs that exceed the accepted threshold [9,35].

It has been argued that certain characteristics of orphan drugs may justify deviating from standard value-for-money criteria [36]. These characteristics may include the severity of the condition and a lack of effective alternative therapies [37].

Uptake curves depend on changes in clinical guidelines, the expected entry of competitive products following registration of a new drug, which may both lower volume units and prices due to competition, and potential other determinants (e.g., expected generic substitutes). There is no single universal ‘uptake curve’ for orphan vs. non-orphan drugs because uptake depends on disease severity, unmet need, competition, and administration route [38]. Non-orphan drugs have much larger patient populations, broader physician adoption curves, and more competition. Uptake is often initially slow but continues to expand steadily from year three onward. Orphan drugs, which are typically developed for severe diseases with a high unmet need, tend to have higher initial uptake in the first years, followed by a plateau as the identifiable patient pool becomes exhausted. Rare diseases are usually treated by a small number of specialists or centers of excellence, and therefore companies can reach most prescribers quickly.

### 3.4. CoC

The CoCs are 9% and 12 for pharmaceutical companies and biotechnology companies, respectively [9]. The investor estimates the CoC based on expectations at the time of investment. The appropriate time horizon for an economic valuation of an R&D project for an innovative drug is 20 years: patent registration occurs in year one, with the end of the patent period at year 20 [9]. The assumption is that the new drug obtains registration at year eight and that the drug will be reimbursed within one year, and consequently, there are eleven years left for actual sales before the patent expires. These assumptions can be weakened in scenario analyses by modeling these moments in time and duration as random variables with given probability distributions.

Double counting of uncertainty for a parameter should be avoided by not including it in the CoC when it is already included in the cash flows (costs and sales). The operational expenses should ideally be incorporated in the numerator (free cash flows) of the present value equation (Equation (1)) and the non-operational uncertainty, e.g., financial expenses, in the denominator (CoC) [9,39]. In the previous sections, we showed that uncertainty due to known higher standard deviations can only be captured in the DCF analysis by transferring the additional uncertainty in cash flows into a higher CoC.

The CoC for biotechnology is an average value with an uncertainty distribution, which means that the CoC may vary based on company-specific features; for example, an important determinant of the CoC is leadership and a track record of previous successful launches [40]. The therapeutic area and competitive environment may have an influence on the risk profile and may lead to a higher CoC for a new high-risk-profile research area, like immunotherapy. The investor may include a premium based on their expert opinion, using the so-called build-up method. An alternative approach is to consider incremental betas for new risky innovative comparable research. The CoC for traditional drug R&D projects may not reflect the CoC for innovative high-risk-profile research. Investors therefore add a variety of premiums to the base rate returned by the CAPM. Sensitivity analyses may be performed by varying the CoC from the lower to the upper limit. In addition, scenario analyses may be performed using different assumptions for the premium for the CoC.

There are different methods to estimate the CoC, for example, rate of return based on pharmaceutical bonds (1–5%) [41], typical government investments (3–7%) [42], the Capital Asset Pricing Model (10–11%) [43], or more flexible models that better account for systematic risk (10–13%) [44].

The company’s own beta is generally not an appropriate measure of the systematic risk of one of its R&D investment projects, which is especially true for large pharmaceutical companies. These large companies have already existing revenues from launched drugs, which reduces the overall beta, while the beta for a risky R&D project should be higher. The beta for small biotech companies with only a limited number of drugs in development and without current sales from other drugs may be a more appropriate estimate of the actual beta for R&D projects.

Another consideration is that the discount rate decreases over time [45]. At the beginning of the R&D process, high fixed obligations must be met before the company can actually start making money. So, the CoC is higher for money invested very early in the process than for money invested later, when the project is nearing market approval. Therefore, early R&D projects have a higher CoC, because they are riskier than later projects [46]. This higher risk of early phases of the R&D project does not result from the probability of failure during the R&D process, as these are diversifiable risks. The real rationale is that operational and financial leverage decrease during the clinical development process, which reduces the risk for the investor. This means that the CoC can be adjusted annually over the years until registration and that a fixed annual CoC can then be applied after market introduction.

While the CoC for a start-up company is quite high, e.g., more than 20%, the CoC becomes already close to the discount rate of a pharmaceutical company, between 8% and 10%, after market launch of the drug [45].

## 4. Alternative Evaluation Method

The alternative evaluation method proposed is the real option methodology that can capture the additional risk through higher standard deviations in economic valuation. This is particularly relevant for the appropriate assessment of the economic value of orphan drugs given their specific uncertainties. Options are particularly attractive when the volatility is high, that is, when there is large uncertainty about future sales and costs, as with orphan drugs [11,12,13]. Real options are especially estimated to add significant value to biotechnology companies, since the industry is characterized by high volatility, relatively long maturity times, and huge upfront investments, all of which have a positive impact on option value.

Real options analysis explicitly recognizes that future value-maximizing decisions will depend on new information that becomes available only after the initial investment has been made for the initial phases of the clinical development program [47,48,49,50,51]. This corresponds to the sequential availability of data regarding the success of the different phases of clinical trials. Hence, real options analysis captures the Value of Information concept, which means that the economic value of an R&D project can be updated after each phase of the clinical program, e.g., after phase I, phase II, or phase III trials, based on the most recent clinical data from the trials. Changes in reimbursement and pricing legislation and corresponding changes in data requirements for national market access applications based on a local pricing and reimbursement scan performed in key countries can also be incorporated. In addition, when a biotechnology company invests in new product development, it has acquired the right but not the obligation to take the product to the market.

## 5. Different Perspectives

We distinguish four types of investors: a large investor with a large portfolio, a small investor in portfolio funds, a small investor in a specific R&D project, and an initial founder of a start-up. The large investor with a large portfolio may diversify the unknown diversifiable risk and may assume average values, unless it is known *a priori* that there are parameter with a different default value and higher standard deviation. The same applies for the small investor in portfolio funds. However, a small investor not investing in large funds but investing in a specific R&D project cannot eliminate risk through diversification and will include this higher risk in the CoC. We may also consider the original owner of the patent (intellectual property), e.g., the scientist who discovered the innovation, who is considering a start-up by investing private money for the initial steps in clinical development. We do not consider the option that a start-up company with private capital, instead of investing in R&D, invests its capital in other financial markets. The rationale is that a biotech company’s core business is innovative drug development instead of being active in financial investments.

## 6. Analysis

### 6.1. Real Option

The economic valuation of an R&D project can be based on the deterministic DCF method when considering a large investor with a portfolio, provided that the unknown diversifiable risks associated with cost, failures, and sales are two-sided. This applies to an R&D project undertaken by an established biotech or pharmaceutical company with backup from revenues from other launched drugs. ‘Unknown’ means that the investor has no information that the mean and SD for the parameters will be different. On the other hand, if there is information on a different mean, the investor can still use the deterministic DCF method, and if there is a difference in SD, this higher risk can be captured in the CoC. Additionally, this investor may be a shareholder in an existing pharma or biotech company starting the R&D project, and a higher spread in NPV increases risk for the investor.

When it concerns an initial R&D project for a start-up company, there is no two-sided risk, and the deterministic DCF method may not be an appropriate valuation method for the investor, especially for the initial founder of a start-up. In this case, the higher spread in NPV increases the potential economic value and therefore reduces the risk for this investor.

Table 1 shows an illustration of the paradox that higher uncertainty for the initial founder in a start-up biotech company increases the NPV. The initial NPV at the time of clinical project initiation is much higher based on a real option approach than the DCF model because it includes partial uncertainty for the initial founder. The economic value increases for both the DCF model and the real options model after every successful phase in the clinical project, but the economic value according to real options analysis increases to a lesser extent due to a reduction in uncertainty after each successful phase. However, the NPV after phase II is much higher according to the real options model ($680 million) than the DCF model ($180 million).

However, if a drug is acquired by a pharmaceutical company after phase II, the one-sided risk becomes a two-sided risk for the large investor, and the pharmaceutical company is only willing to pay the lower value of $1.8 million, based on the DCF model. If the original founder is risk-averse and postpones the acquisition until after the product launch, the NPV according to the real options model ($990 million) remains the true economic value for the founder.

Further in-depth analysis of the application of real options models for clinical R&D project valuation is beyond the scope of this paper.

### 6.2. Perspective

There is also an important difference in capturing uncertainty for an initial founder of a start-up compared with the shareholder in an established biotech or pharmaceutical company. A start-up only has one (or a few) innovative products in development without any simultaneous sales of launched products to offset the R&D costs. The R&D costs must be captured by future sales and not by sales from other products in accordance with economic valuation theory. However, from an accounting point of view, the current liquidity (income statement) benefits from revenues generated by sales from other products, which offsets the simultaneous costs of the R&D project.

**Figure 1:**
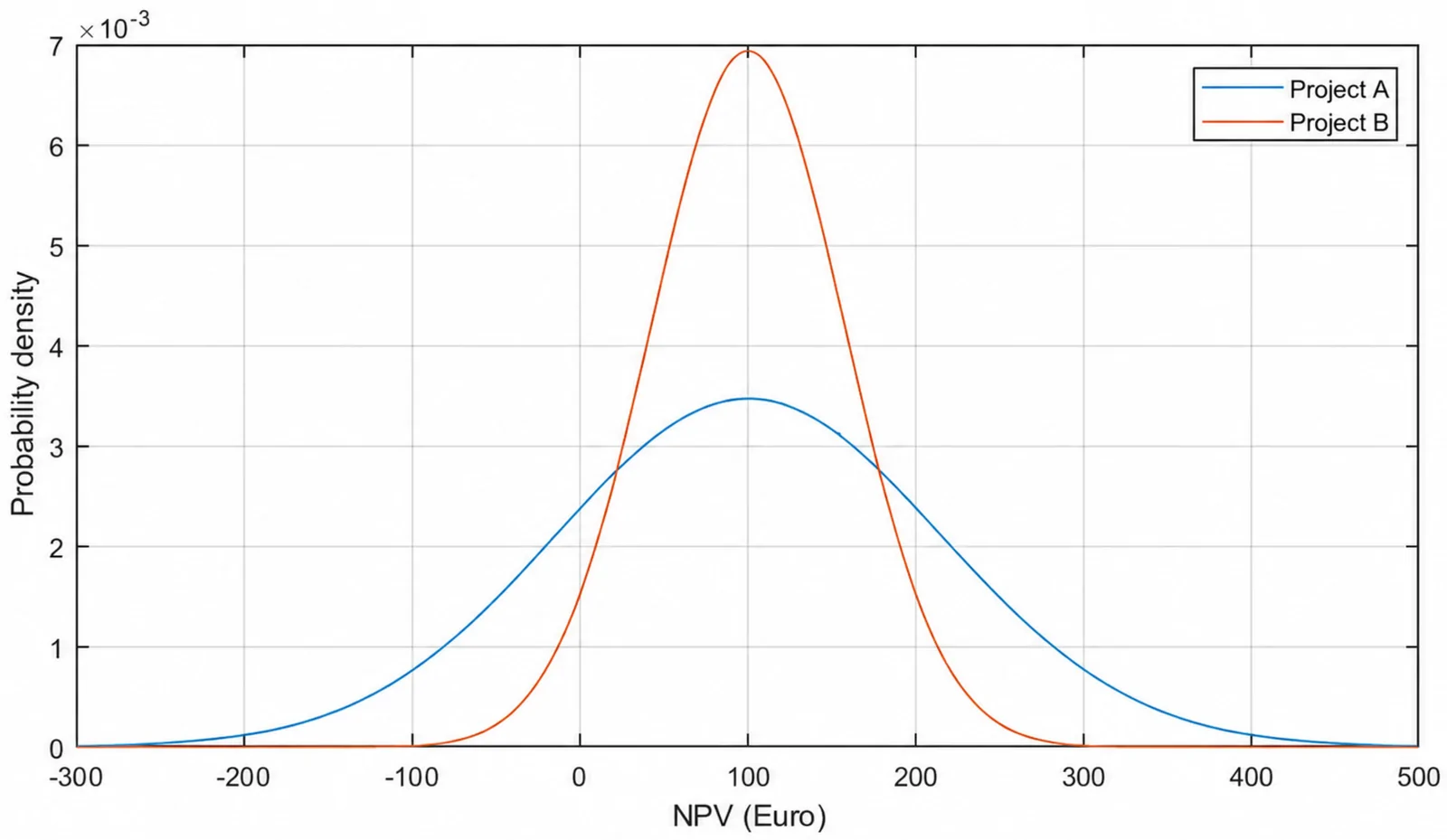
Distribution of NPV-start-up.

Figure 1 shows the two possible distributions of NPV for a start-up company:

- Project A: The average NPV is €100, with 95% distribution from −€130 to + €330.
- Project B: The average NPV is €100, with 95% distribution from −€15 to + €215.

The initial investment for both cases is €50, which is the maximum possible loss for the investor. Therefore, the investor will not suffer more from the potential loss of €130 in Project A than the potential loss of €15 in Project B in the worst-case scenario. However, the investor may benefit from a higher potential gain of €330 in Project A than €215 in Project B. Hence, the potential loss is €50 in Project A and Project B because of limited liability, the founder cannot lose more than the €50 invested, but the potential gain is €115 higher (€330 − €215) for Project B than Project A. This example shows that the DCF analysis, which is based on a diversifiable risk, yields an NPV that does not reflect the actual economic value for the initial founder. The second observation is the paradox that higher uncertainty for an initial founder in a start-up biotech company increases the NPV because of the partially one-sided risk.

**Figure 2:**
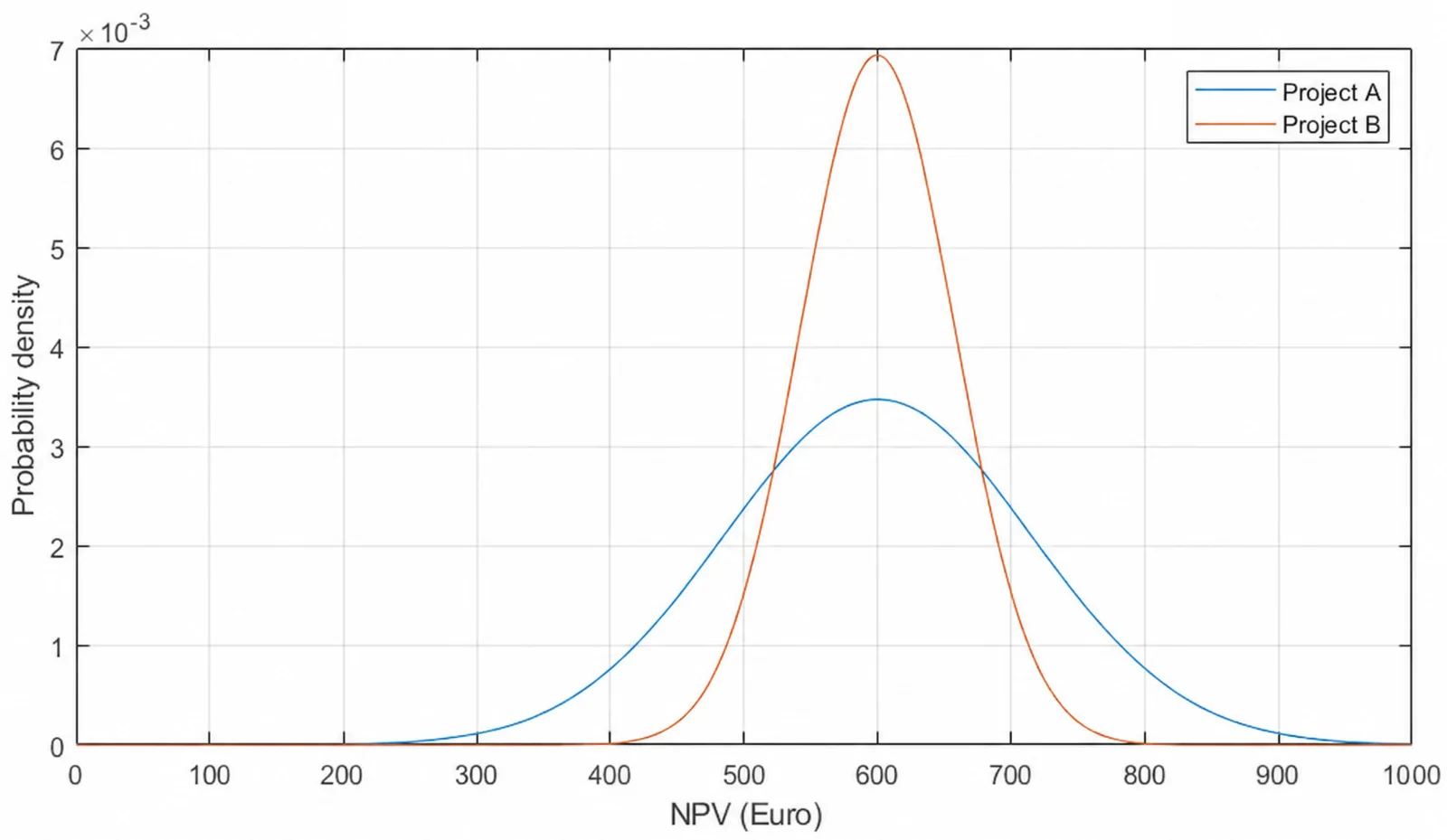
Distribution of NPV—established company.

Figure 2 shows the impact of uncertainty/risk for a shareholder in an established biotech or pharmaceutical company for the same R&D Projects A and B: the difference is that the NPV of this R&D project is added to the NPV of other R&D projects and sales of existing drugs, e.g., NPV of €500.

In this case, the shareholder has a two-sided risk, which means that Case A is riskier than Case B and the shareholder will choose Case B if we assume the shareholder is risk-averse. For Case A the best-case value = €500 + €330 = €830 and worst-case value = €500 − €130 = €370. The largest possible reduction in value relative to the expected value is therefore €600−€370 = €230. Similarly for case B €600 − €485 = €115. The potential maximum loss is no longer the investment of €50 for Case A and Case B, but €230 for Case A and €115 for Case B.

## 7. Discussion

The common approach to economic valuation is based on the concept of discounted cash flow. Discounted cash flow is based on the present value equation, which calculates the time value of money and compounding returns. The discounted cash flow method is based on a deterministic approach, which means that the economic value, e.g., NPV, does not reflect any spread in the statistical distributions of the parameters and any other non-statistical uncertainty, e.g., selected data sources or assumptions. The NPV for innovative drugs may be lower than for traditional drugs because of higher cost, higher probabilities of failure in R&D, and lower sales, which is captured in the deterministic outcome of DCF analysis. These parameters will also have a higher standard deviation due to higher uncertainty in the expectations for innovative drugs, like immunotherapy and gene therapy. This uncertainty seems diversifiable for the investor by spreading the uncertainty over a large portfolio of R&D investments, but when an investor is risk-averse, a project with a lower standard deviation is preferred over a project with a higher standard deviation when the projects have similar deterministic NPVs.

The examples in this paper showed that the NPV for starting an R&D project is higher than the same R&D project for a shareholder in an existing company. This economic value can only be captured if the innovative drug is also actually launched by the start-up. However, if the start-up company is acquired during the R&D development phases, the economic value drops after the acquisition because the one-sided risk becomes a two-sided risk, leading to a conversion of the impact of risk. This drop in economic value is higher when there is a large spread in risk. Consequently, the economic value for a start-up R&D project should include the probability of an acquisition during the clinical development stage. If we assume most start-ups are acquired during the development phase, this means that the economic value is reflected by the discounted cash flow method using two-sided risk. A more accurate assessment would be based on weighting the economic value by the probabilities of acquisition and non-acquisition during the development phase until market access (registration and reimbursement).

An acquisition may also occur after market access, but the impact of a late acquisition is much lower because the spread in cash flows resulting from the spread in sales is much smaller than the spread in early cash flows due to development costs, R&D costs, and, in particular, the probabilities of clinical failures. Consequently, the initial founder of a start-up could capture the maximum value due to one-sided risk by postponing the acquisition until after registration and market access. Hence, the funding of the development phase is critical for initial investors. It may be more profitable to invest more in funding (higher loans or giving more shares to external investors) and therefore capture the much higher economic value based on the one-sided risk by avoiding the financial need for acquisition before R&D phase finalization.

Clinical R&D project timing reflects an additional uncertainty in economic valuation that is not captured in the DCF method. The pay-off for an investment in a clinical R&D project requires the investor to wait for initial sales after the possible successful completion of the clinical program, which may take eight years. The discounted cash flow model captures time using discounting in the CoC. Nevertheless, an investor will prefer a project with sales within one year over another project with sales after eight years if both projects have the same NPV.

However, once the investor has been investing in start-ups for more than eight years, every annual investment from the latest investment is rewarded by initial sales of the project from eight years ago. A long-term investor may consequently require a lower CoC than an initial investor, which means that economic value based on the discounted cash flow method is different for a long-term investor and a new investor.

This research suggests that the real options model may be preferable to the DCF model for investment decisions in orphan drug development because it more effectively captures the high level of uncertainty involved. We considered the perspective of four types of investors: a large investor with a large portfolio, a small investor in portfolio funds, a small investor in a specific R&D project, and an initial founder of a start-up. However, there is much greater heterogeneity among investor types, including angel investors, seed investors, venture capital funds, private equity firms, institutional investors, and public market investors. Public authorities frequently act as investors of first resort, funding high-risk innovation projects that private investors are not always willing to support. DCF and NPV are usually embedded within a broader framework because many public investments generate social, environmental, and economic benefits that do not translate directly into cash flows. Examples are cost-effectiveness, budget impact, unmet medical need, equity considerations, and broader social values.

In addition, each stage of development tends to attract a specific type of investor; for example, angel investors are typically involved in the early stages, followed sequentially by venture capital funds, private equity firms, and later public market investors.

The Black–Scholes model was the first and most well-known application of evaluating options [52,53,54,55,56,57]. This concept was broadly adopted across various fields between 1999 and 2015, particularly in the pharmaceutical sector [58,59]. Five variables determine the value of a simple financial option according to Black and Scholes model: an underlying S, an exercise price X, the time to maturity T, the volatility (J of S), and the risk-free interest rate [10]. However, some of the assumptions of Black and Scholes model are violated due to the fact that we are concerned with incomplete markets in this particular application; it provides a relatively accurate value, but only at the end of the research and development phase. Several other approaches have been developed over the years to calculate the real options value of an investment [60,61,62,63,64]. The compound option analogy reflects the value of the growth during sequential phases in clinical development more accurately [10].

These previous studies have demonstrated the advantages of real options for pharmaceutical valuation. Our contribution extends this literature by explicitly distinguishing investor types and showing that identical uncertainty may have different economic implications depending on whether risks are diversifiable. Rather than replacing DCF, real options should be viewed as complementary. DCF remains appropriate for estimating expected cash flows, while real options capture the strategic flexibility embedded in sequential R&D decisions.

There are also practical implications for both investors and biotechnology companies. Investors may use real options analysis alongside conventional DCF models to evaluate whether flexibility in development decisions creates additional value. For biotechnology companies, particularly start-ups developing orphan drugs, the approach provides a framework for determining optimal financing strategies, milestone planning, and timing of licensing or acquisition discussions.

The real options approach also has several limitations. One major challenge is the estimation of volatility, particularly because many of the model parameters are based on forecasts (e.g., size of the market, market uptake, and clinical failure rates) rather than observed data. Estimates of mean values rely on assumptions and historical data that are difficult to validate. Furthermore, estimating the standard deviations used to measure volatility may be even more challenging, introducing additional uncertainty into the analysis.

Further validation may be the next step. For example, we may test whether investors in treatments for rare diseases indeed adopt a different approach to uncertainty in their discounted cash flow (DCF) calculations compared with investors in treatments for non-rare diseases. Such differences could imply that the risk profile and NPV characteristics differ systematically between the rare-disease and non-rare-disease domains.

Further research may also focus on the optimal treatment of uncertainty in both discounted cash flow (DCF) and real options analysis, as well as on the methodological differences between these two approaches. For example, the use of probabilistic sensitivity analysis (PSA) may be valuable, as it can incorporate probability distributions into the mean net present value (NPV) estimated in DCF analyses.

The findings suggest that reimbursement uncertainty contributes substantially to investment risk. In a previous study, we demonstrated that investor uncertainty is a key determinant of drug prices [37]. Consequently, regulatory initiatives that enhance the predictability of pricing and reimbursement decisions may reduce uncertainty, ultimately leading to lower drug prices.

## 8. Summary

In this paper, we explored how different investor types perceive and manage uncertainty in the valuation of innovative drug development. By moving beyond conventional models, we aimed to provide a more nuanced understanding of value creation in high-risk, high-reward medical innovation environments. We described different types of uncertainty and explored different approaches to dealing with uncertainty in the economic valuation of medical innovation in orphan diseases. The DCF approach may not be suitable for an investor in innovative drugs due to the high degree of uncertainty.

We examined the limitations of the DCF approach in capturing the true economic value of high-risk, high-reward innovations. From the perspective of large investors with diversified portfolios, many of these risks are considered diversifiable and are therefore reflected in the aggregate portfolio’s expected return. In contrast, small investors and founders of start-up biotech firms typically lack the ability to diversify risk across multiple projects. For these stakeholders, the consequences of failure are more direct, and the strategic timing of decisions such as the sale of a company or the continuation of development becomes a crucial factor in value creation. A real options model may be applied to capture the uncertainty in economic valuation of early-phase drug development, which provides a more realistic valuation by incorporating uncertainty as a value driver. This alternative approach shows how uncertainty, often viewed as a liability, can paradoxically enhance value under specific investment conditions in orphan drug development.

## Figures and Tables

**Table 1:** Comparison of DCF model and real options model.

Phase	Economic Value (NPV)	
	DCF	Real Options
start	$31.6 million	$320 million
>Phase I	$46.8 million	$420 million
>Phase II	$180 million	$680 million
>Phase III	$390 million	$940 million
>Phase IV	$440 million	$990 million

## Data Availability

The original contributions presented in this study are included in the article. Further inquiries can be directed to the corresponding author.
